# Study on the Viability of Canine Nose Pattern as a Unique Biometric Marker

**DOI:** 10.3390/ani11123372

**Published:** 2021-11-25

**Authors:** Hyeong In Choi, Mu-Young Kim, Hun-Young Yoon, Sungjin Lee, Stephanie Sujin Choi, Chang Yong Han, Hwan Pyo Moon, Changhyun Byun, Song-Hwa Kwon

**Affiliations:** 1 Department of Mathematics, Seoul National University, Seoul 08826, Korea; hichoi@snu.ac.kr; 2 iSciLab Corporation, Seoul 08791, Korea; choistephanies@iscilab.com (S.S.C.); cbyun@iscilab.com (C.B.); 3 Department of Veterinary Surgery, College of Veterinary Medicine, Konkuk University, Seoul 05029, Korea; zypo@konkuk.ac.kr (M.-Y.K.); yoonh@konkuk.ac.kr (H.-Y.Y.); 4 Department of Mathematics, Daejin University, Pocheon 11159, Korea; hyper@daejin.ac.kr; 5 Department of Applied Mathematics, Kyung Hee University, Yongin 17104, Korea; cyhan@khu.ac.kr; 6 Department of Mathematics, Dongguk University, Seoul 04620, Korea; hpmoon@dongguk.edu; 7 Department of Mathematics, The Catholic University of Korea, Bucheon 14662, Korea

**Keywords:** canine, dog, nose, nose pattern, nose print, biometrics, biometric marker, template, gabor transform, hamming distance

## Abstract

**Simple Summary:**

This paper shows that the canine nose pattern, which is an interlocking pattern of beads and grooves on a dog’s nose, is unique to each individual dog. For this purpose, the nose images of 60 dogs were collected at three separate times, each roughly three to four months apart. This longitudinal cohort study was designed to ensure the diversity of data, wherein dogs of diverse age, gender, and breed are well represented in the dataset. In this study, the nose patterns of these dogs were examined visually and by a biometric algorithm to determine the uniqueness of the canine nose pattern. It was found that the canine nose pattern remains invariant through the passage of time during the observation period; and that the canine nose pattern is indeed unique to each dog. Our finding confirms and enhances the claims of earlier works by others that the canine nose pattern is unique to each animal and serves as a unique biometric marker. For further study, this dataset was augmented by adding to it the nose images of 10 beagle dogs taken once every month in a ten-month period to create an enlarged dataset of 278 images of 70 dogs of 19 breeds. The study with this enlarged dataset also leads to the same conclusion.

**Abstract:**

The uniqueness of the canine nose pattern was studied. A total of 180 nose images of 60 dogs of diverse age, gender, and breed were collected. The canine nose patterns in these images were examined visually and by a biometric algorithm. It was found that the canine nose pattern remains invariant regardless of when the image is taken; and that the canine nose pattern is indeed unique to each dog. The same study was also performed on an enlarged dataset of 278 nose images of 70 dogs of 19 breeds. The study of the enlarged dataset also leads to the same conclusion. The result of this paper confirms and enhances the claims of earlier works by others that the canine nose pattern is indeed unique to each animal and serves as a unique biometric marker.

## 1. Introduction

In this paper, we studied the canine nose pattern at the tip of a dog’s nose, and examined whether the pattern there is unique to each individual dog and is usable as a biometric marker.

**Figure 1:**
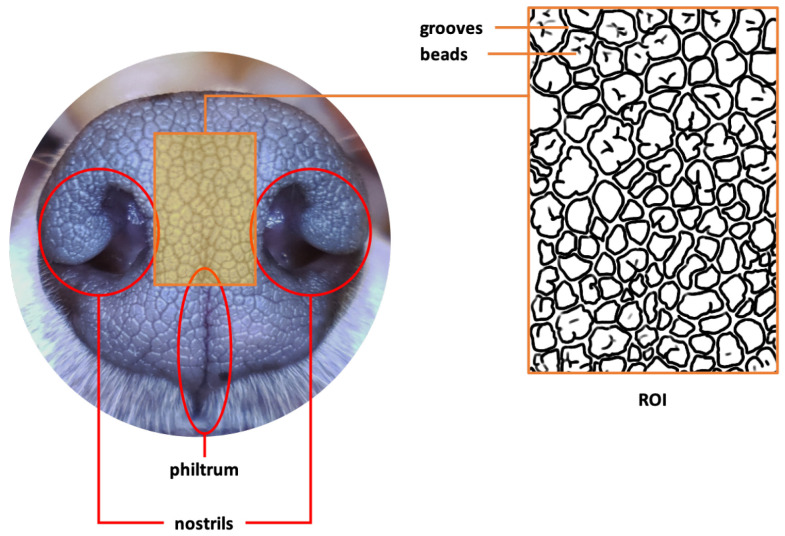
Canine nose and nose pattern in ROI (replicated with permission by the copyright holders).

The region to which we focused our attention is the part of a dog’s nose between and around the right and left nostrils, including the area around the philtrum and extending slightly up above the nostrils. This region at the tip of a dog’s nose, called the *Region of Interest* (*ROI*), has a very complex folding pattern of skin consisting of obtruded regions, called *beads*, and sunken and narrow ribbon-like regions between the beads, called *grooves*. This complex interlocking pattern of beads and grooves is called the *canine nose pattern*. The ROI and the interlocking of the beads and grooves are illustrated in Figure 1, which is taken from [1] with the copyright holders’ permission.

There is a sizable body of literature–academic or popular–claiming the uniqueness of the nose pattern of certain animals. As far as we know, the earliest academic work on the uniqueness of the canine nose pattern was done by Horning et al. [2] in 1926, in which they found that the canine nose pattern can be used to distinguish between individual dogs. This result is also cited in the well-known textbook, Miller’s Anatomy of the Dog, 4th Edition by Evans and de Lahunta [3]. Budras et al. [4], in their textbook, Anatomy of the Dog: An Illustrated Text, Fifth Edition, claim that “The dermis forms distinct papillae. The epidermis is strikingly thin, and its superficial, cornified layer (stratum corneum) consists of hard ‘horn’ (hard cornified epidermis) that exhibits a polygonal pattern. The surface pattern is individually specific and for this reason serves to identify the individual animal”. There is a proliferation of popular articles claiming the uniqueness of the canine nose pattern. For instance, see [5,6].

Dogs are not the only species with a unique nose pattern. Members of canids like dogs and wolves, bovids like cattle, felids like cats, and cervids like deer also have individually unique nose patterns. Due to the similarities in the biological traits and origins of the nose organ of such species, a positive result on one species is a very good indication that the same may be true for the others. As such, it is profitable to go over prior results on cattle nose pattern identification.

In 1922, Petersen [7] manually took the nose prints of more than 350 animals and observed that no two were found to be alike, and thus the nose prints can be used to distinguish different animals.

Since then, there has been a proliferation of works on developing cattle nose identification *algorithms*. For this, one may consult the article by Awad [8] for a good overview. There are many similar studies. Barry et al. [9] used the techniques of principal component analysis and Euclidean distance classifier to printed and digital image sets of a small number of cattle and found their eigenvector method exhibits promising results.

There are other results based on various image processing techniques such as Speed-Up Robust Features Approach (SURF), Scale Invariant Feature Transform (SIFT), and Local Binary Pattern (LBP). U-SURF, a variant of the Speed-Up Robust Features Approach (SURF), was used by Noviyanto and Arymurthy [10] for a good result. The same authors [11] later improved it using SIFT to match key-points on the muzzle pattern lifted on paper. Similarly, Awad et al. [12,13] also used SIFT and the key-point match method. A texture classification method using LPB was employed by Tharwat et al. [14] to extract local invariant features from muzzle print images, and then different classifiers including Nearest Neighbor, Naive Bayes, SVM and KNN were applied for cattle identification. Tharwat et al. [15] used Gabor filter-based feature extraction and machine learning. In [15], the Gabor features extracted from three different scales of muzzle print images were used by the SVM classifier, while in [16] three different classifiers (support vector machine—SVM, k-nearest neighbor, and minimum distance classifier) were used. They found that in general the so-called feature fusion-based models achieve better accuracy.

Machine learning and deep learning models are gaining popularity. For example, Hadad et al. [17] studied muzzle print identification using Artificial Neural Network (ANN) and K-nearest neighbor classifier (KNN); and Kumar et al. [18] also used a deep learning approach. Gaber et al. [19] used Weber Local Descriptor (WLD) to extract features from cattle muzzle print images, and then employed the AdaBoost classifier to identify heads of cattle from their WLD features. Similarly, Awad and Hassaballah [20] used the bag-of-visual-words (BoVW) approach with the features extracted by SURF and the maximally stable extremal regions (MSER).

Additionally, Kumar et al. [21] developed algorithms that closely resemble fingerprint minutiae matching. In [21], they used the SIFT algorithm to detect interest points in a cattle image and then used the FLANN (Fast Library for Approximate Nearest Neighbor) matching algorithm for identification.

All of the above results concern cattle nose pattern identification. With regards to dogs, the literature is rather scant. Kumar and Singh [22] examined the performance of existing face recognition algorithms as applied to dogs. Jang et al. [23] developed a three-step algorithmic procedure consisting first of image preprocessing using resizing and histogram equalization; then feature extraction using several image processing techniques such as SIFT, SURF, BRISK and ORB; and finally matching using FLANN for SIFT and SURF, and hamming distance for BRISK and ORB. After some post-processing they examined their method on 55 dog muzzle pattern images acquired from 11 dogs and 990 images augmented by image deformation (i.e., angle, illumination, noise, affine transform). The best Equal Error Rate (EER) of the proposed method was 0.35%, and ORB was found to be the most appropriate for dog muzzle pattern recognition.

It must be emphasized that most, if not all, of the above works, except for the fundamental works like those of Horning et al. [2] and Petersen [7], are concerned with developing as accurate an *algorithm* as possible, thereby tacitly presuming the uniqueness of the animal nose pattern as an underlying assumption. So, when it comes to the fundamental question of the *intrinsic biological uniqueness* of the nose pattern, it is fair to say that the evidence is not fully corroborated. For example, most of the algorithms entail some error in one form or another in identification. If an error of an algorithm occurs, it is not clear if it is due to bad technology, or because some two animals have sufficiently similar nose patterns that are not distinguishable. Unless an algorithm shows a 100% accuracy, the algorithm itself cannot be taken as evidence for intrinsic biological uniqueness.

We believe that this fundamental question must be resolved as a foundation for good algorithmic development; hence, the purpose of this paper is to resolve this question by verifying that the canine nose pattern is indeed an intrinsic and unique biological property of a dog.

There is another important issue to be clarified, which is how the images are captured. Traditionally, the bovine (and canine) nose pattern was obtained using a manual imprinting method. This was done by spreading a small amount of ink over an animal’s nose, pressing onto which a paper or soft cloth to lift the nose pattern impression, or nose print. Essentially, the same imprinting mechanism was utilized for image capture by Horning et al. [2], Petersen [7] and many others. Since this is an imprinting method, the term “nose print” or “muzzle print” had been commonly used. We, however, use the term “nose pattern” to emphasize the fact that what really matters is not the “print” of the nose per se, but the actual nose *pattern* as an intrinsic property of an animal.

As for the actual practice of utilizing a dog’s nose print as a means of registration and management of a dog’s identity, the most well-known example is that of the Canadian Kennel Club accepting canine nose prints as proof of identity since 1938 [24,25]. What was to be presented to the Canadian Kennel Club was the actual print of a dog’s nose (nose print) imprinted on a paper with ink.

However, this manual muzzle imprinting method is too cumbersome to be used as a practical means of identifying animals in a live operation. It takes time and effort to hold the subject animal still during the imprinting process; and moisture on the animal’s nose may produce a smeared image.

Moreover, the imprint method—or, for that matter, any contact method—may cause problems. As Davis [6] puts it: “The nose consists of fragile mucus glands, so any drying or agitation could hurt. This all being said, you probably shouldn’t touch it. Your hands carry a lot of bacteria, oils, and dirt. Best case, you cloud their sense of smell with the oils from your hands”.

With the modern digital camera, however, such mechanical or manual imprinting method is no longer favored. Instead, one uses a smart phone camera to capture the nose image without making any contact with the animal’s nose. The nose images collected in this paper were all captured by smart phone cameras.

For these reasons, the Canadian Kennel Club no longer accepts nose “prints”; they now rely on microchips inserted into the animal’s body. In fact, microchips have become the standard in canine identity management. However, microchips are known to have some side effects to animals in certain cases as observed in various studies: cancers [26,27]; and granulomatous inflammatory response [28]. Similarly, cancers are reported for cats [29,30] and rodents [31,32,33]. Other complications include: various spinal cord injuries [34,35]; paralysis [36] and migration of the microchip to the brainstem [37]. One may consult the paper by Swift [38] that keeps track of adverse reactions to microchips.

For the sake of fairness, it should be noted that such reported side effects due to microchip insertion are rather rare. Nonetheless, the risk acts as a psychological barrier to accepting microchip insertion for some people. Many such people would prefer for their animals the non-invasive method that canine nose biometrics provides. In this sense, canine nose biometrics may have its place as a complementary means of identification to microchip insertion. This will help contribute to improving animal welfare.

The ultimate goal of this paper is to show that the canine nose pattern is unique to each individual dog. This claim is not new. It goes all the way back to Horning et al. [2]. Since then there have been many papers studying the canine nose print (pattern). However, as we have expounded above, there are not that many works that actually examine the uniqueness of the canine nose pattern for its own sake. Except for a few fundamental works like those of Horning et al. [2] and Petersen [7], most of the prior works are about developing as accurate a practical algorithm as possible while tacitly presuming the uniqueness of the animal nose pattern as an underlying assumption. Thus, it is still fair to say that the question of the uniqueness of the canine nose pattern has not been fully resolved.

A recent work by Choi et al. [1] addresses exactly the same issue. The focus of their study is if and when the canine nose pattern is fully formed; and if this nose pattern stays invariant throughout a dog’s life. For this purpose, they collected the nose images of ten beagle dogs for the ten-month period starting from the second mensiversary and ending at the eleventh mensiversary; examined the nose patterns in these images visually and by a biometric algorithm; and concluded that the canine nose patterns of these beagles are fully formed at the second mensiversary and remains invariant until the eleventh mensiversary. Their result is more interesting in the sense that these puppies are siblings. However, their study has some limitations. First, only one breed is present in the experiment: namely, the beagle. Second, the number of dogs involved is small: namely, 10. Third, the dogs are all of the same age, so that it does not allow observation of a diverse age group.

The research of this paper was designed to overcome such shortcomings in [1]. Special care was taken to ensure the diversity of data so that dogs of diverse age, gender, and breed are well represented in the dataset as well as a larger sample size.

The nose images of 60 dogs of 18 breeds were collected at three separate times, each roughly three to four months apart. Each dog participating in the data collection was required to come back three times during the data collection period so that this dataset has the character of a cohort dataset as well as a longitudinal dataset. For further study, this dataset was augmented by adding to it the images used in the study of [1] with the authors’ permission. This enlarged dataset has 278 images of 70 dogs of 19 breeds.

In this study, the nose patterns of these dogs were examined visually and by a biometric algorithm to determine the uniqueness of the canine nose pattern. It was found that the canine nose pattern remains invariant through the passage of time during the observation period; and that the canine nose pattern is indeed unique to each dog.

Our finding confirms and enhances the claims of earlier works by others that the canine nose pattern is unique to each animal and serves as a unique biometric marker.

This paper should be considered as a work complementing that of Choi et al. [1]. We believe both of them taken together provide even more convincing evidence that the canine nose pattern is indeed unique to each individual dog.

## 2. Materials and Methods

### 2.1. Data Collection

The data collection was done using the back cameras of two Samsung smart phones: one is Galaxy S9+ (model SM-G965N) and the other is Galaxy S9 (model SM-G960N). In order to ensure the diversity of data, the nose images of 60 dogs of 18 different breeds were collected. They were taken at three separate times, each roughly three to four months apart. Each dog participating in the data collection was required to come back three times during the data collection period so that this dataset has the character of a cohort dataset as well as a longitudinal dataset. This enabled us to do a good *longitudinal cohort study*.

The details of the data collection are summarized in Table 1. The gender and age distribution is sufficiently varied to reflect the wide variation in the existing population of dogs.

**Figure A1:**
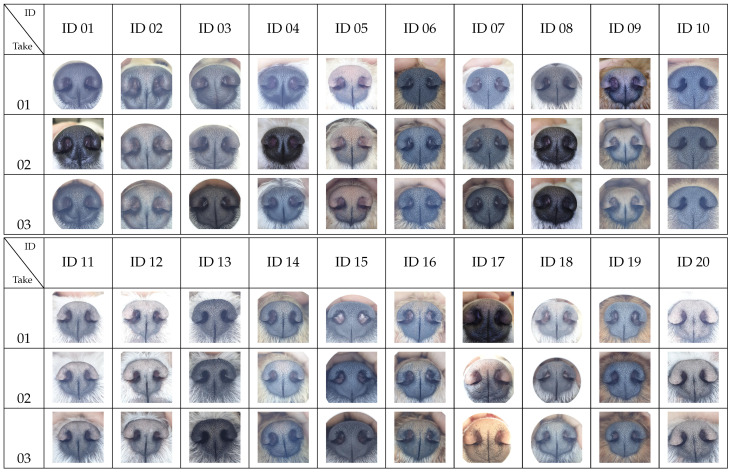

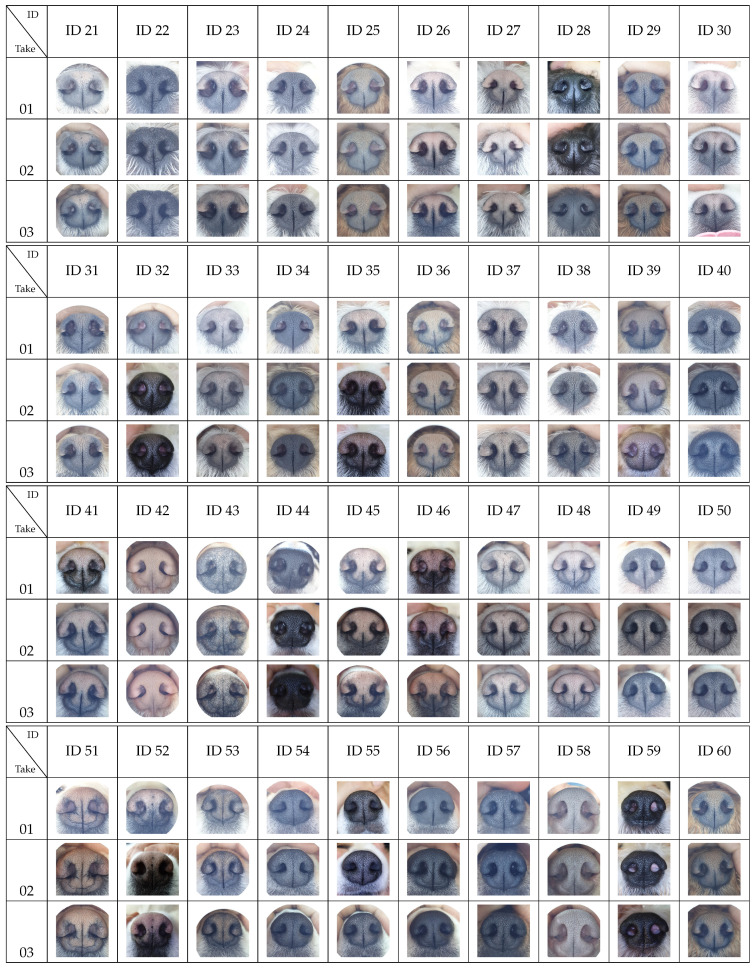
All images.

All of the 180 dog nose images are shown in Figure A1. There are 60 IDs (dogs); the three vertical columns for each ID represent three images of the same dog taken at three different times as described in Table 1.

For further study, this dataset was augmented by adding to it the images used in the study of [1] with the authors’ permission. The enlarged dataset has 278 images of 70 dogs of 19 breeds.

### 2.2. Method of Comparison

The uniqueness of the canine nose pattern can be checked in two ways. One is by direct visual examination and the other by algorithmic verification. For algorithmic verification, we present two results obtained using two sets of data. The first used the 180 images in Figure A1 as described in Table 1. The second used a combined image set of the 180 images in Figure A1 and 98 images collected for the work by Choi et al. [1], for a total of 278 images of 70 dogs.

**Figure 2:**
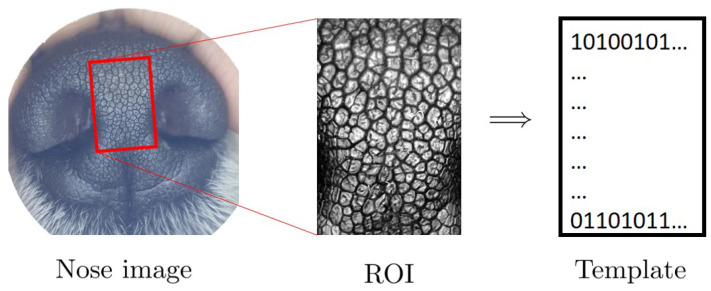
Schematics of template creation.

**Figure 3:**
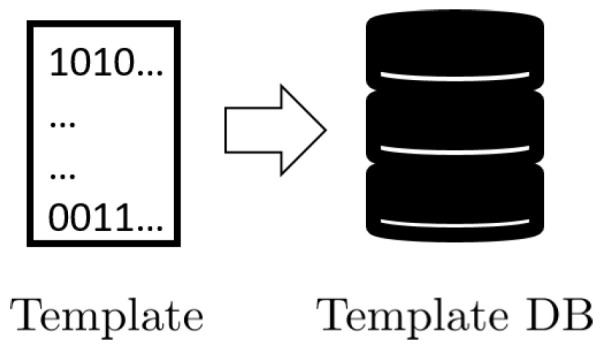
Schematics of template storing.

**Figure 4:**
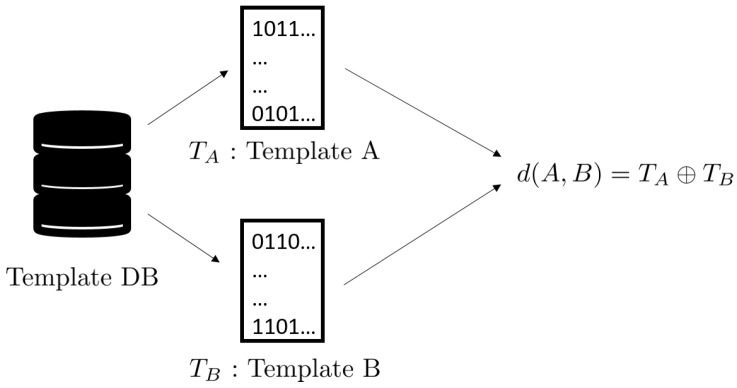
Schematics of template matching.

The algorithmic verification method can be outlined as follows. The first step is the creation of a template from each nose image. Figure 2 shows how it is done. In the left is a nose image of a dog; the middle shows the ROI as in Figure 1; and the right is a biometric template. A biometric template, or in short, a template, is created by applying the well-known Gabor transform. A template is a rectangular array of 0s and 1s whose value at the location $\left(x_{0},y_{0}\right)$ is determined by the sign of the value of Gabor transform at the location $\left(x_{0},y_{0}\right)$. For more details, see [39,40]. Once templates are created, they are stored in a database, the Template DB, as shown in Figure 3. The final step is matching. Figure 4 shows the schematics of template matching. Suppose there are two templates, $T_{A}$ created from the ROI of nose image A, and Template $T_{B}$ from the ROI of nose image B. These two templates, $T_{A}$ and $T_{B}$, are then compared using the well-known Hamming distance [39,40]. The resulting Hamming distance is called the matching distance, and is denoted by $d\left(A,B\right)=T_{A}⊕T_{B}$. By abuse of language, this matching distance is usually called the matching distance between the two nose images A and B, although technically the matching distance computation involves only the ROI of a nose image. This matching distance measures how similar these two templates, and hence the two nose images, are. In general, the smaller the matching distance is, the more similar the two nose images are; and the greater the matching distance, the more dissimilar the two nose images.

When two nose images of the same dog are compared, it is called a *genuine comparison*, and the resulting distance is called the *genuine distance*. Similarly, when two nose images of different dogs are compared, it is called an *impostor comparison* and the resulting distance is called the *impostor distance*. Thus, a good algorithm must exhibit small genuine distances and big impostor distances.

## 3. Results

### 3.1. Visual Examination

**Figure A2:**
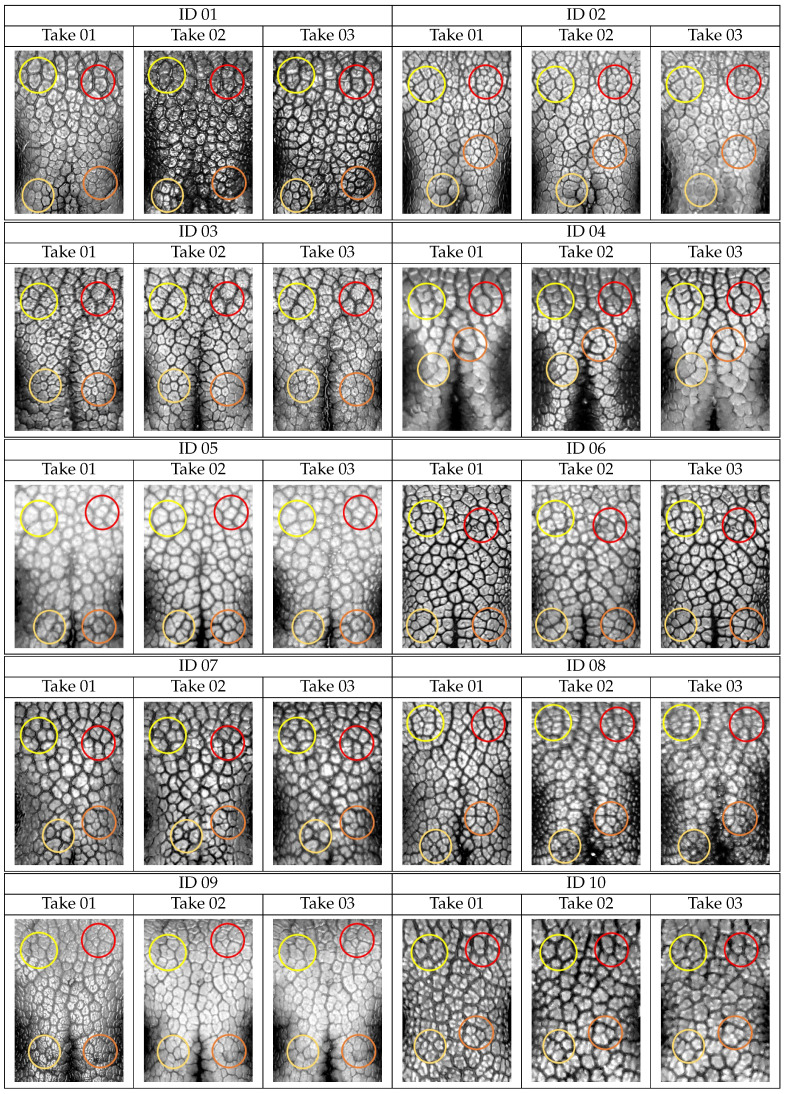

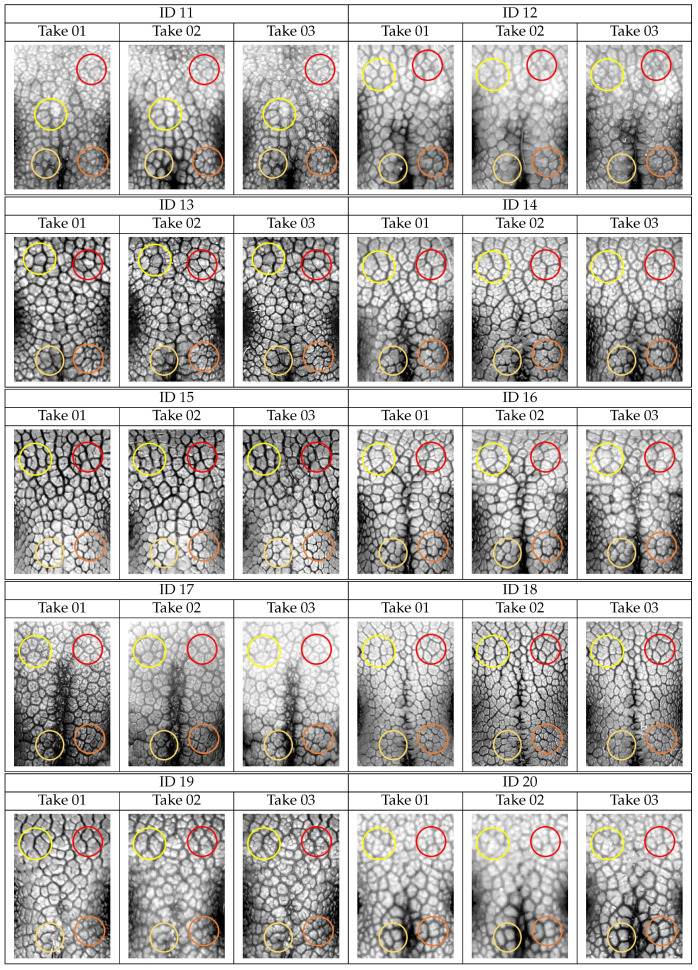

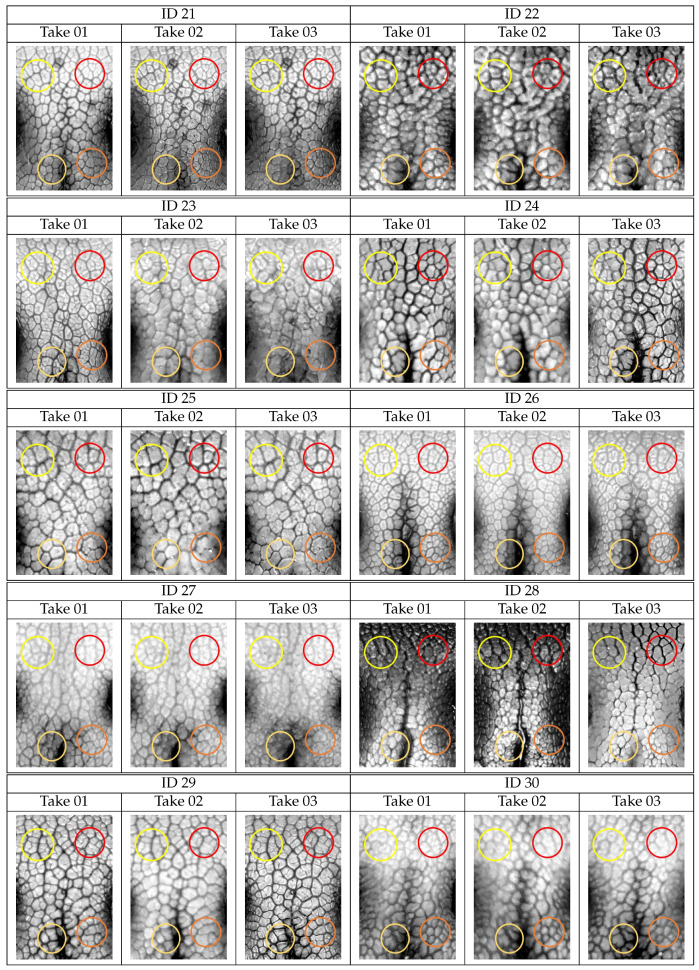

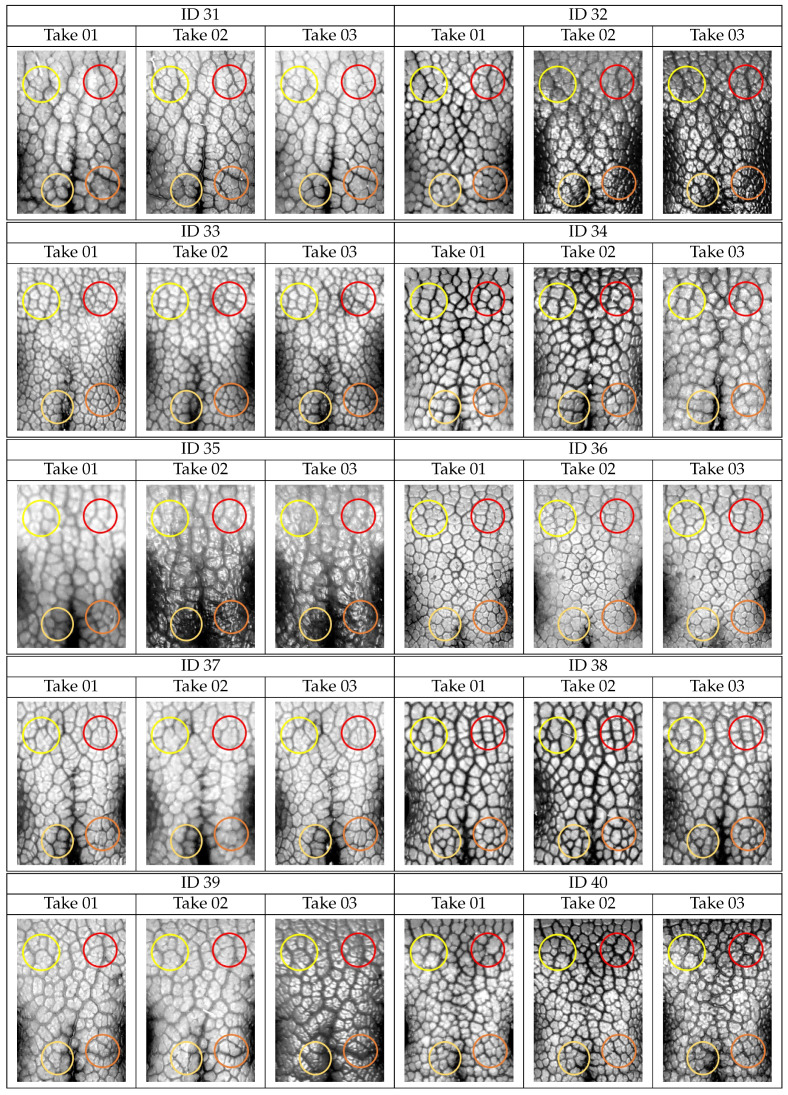

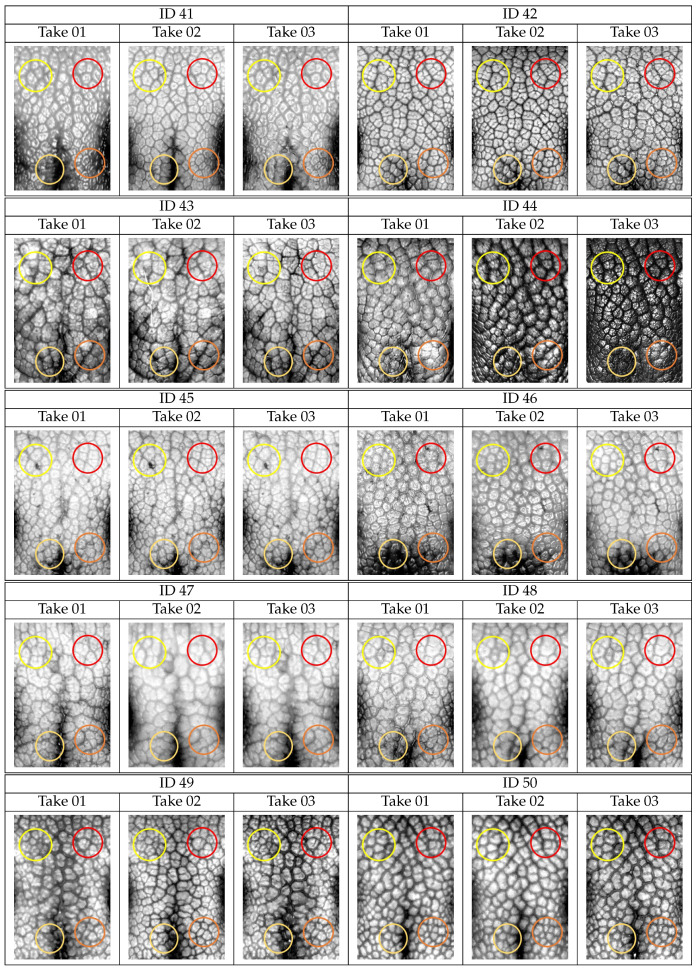

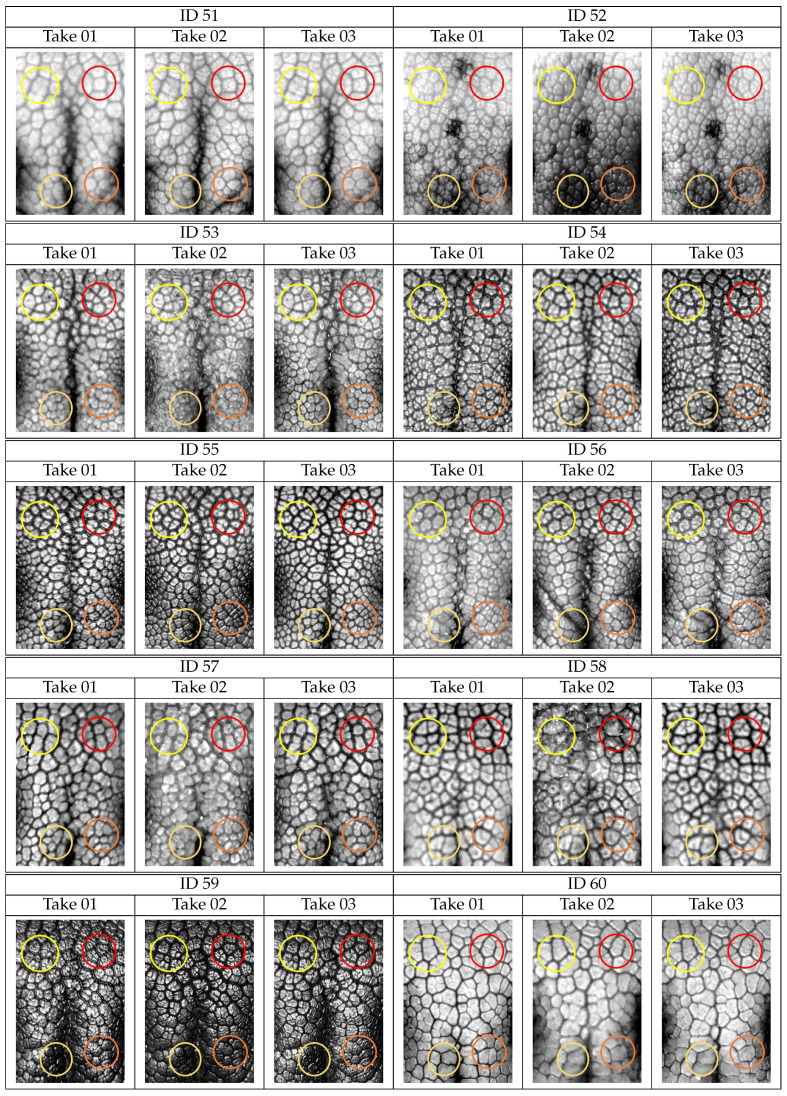
ROI of each image; the same pattern repeats across the three takes of the same ID.

Figure A2 shows the ROIs of the total of 180 nose images of 60 dogs taken three times during the data collection period. For each of the 60 IDs, there are three images described as Takes 01, 02, and 03 representing the three different dates at which the nose images were taken as described in Table 1.

For example, let us examine the three images of Takes 01, 02, and 03 of ID 06 in Figure A2. It is clearly seen that the pattern in the top left yellow oval area remains more or less the same for all three takes, and the other-colored ovals show the same invariance of pattern. In examining other IDs, the same invariance of pattern can be observed. From these observations, one can conclude that indeed a dog’s nose pattern remains invariant.

Next, let us compare the nose patterns of different dogs. For example, compare images of ID 06 and ID 08. Note that the relative locations of the four ovals in the two sets of images are different; and the patterns in the regions within the ovals are also different. Upon closer examination of any pair of images of two different dogs, one can easily see that a similar difference persists in all pairs of nose images from different dogs. This indicates that indeed the nose pattern is unique to each individual dog.

A word of caution is in order. The ovals in no way are something like a landmark or minutiae in a fingerprint. They are purely visual aids overlaid on the image simply to help the reader better discern the canine nose pattern. When it comes to judging the similarities and dissimilarities of the canine nose pattern, the *whole* ROI image has to be examined.

### 3.2. Algorithmic Verification I

The visual examination method in Section 3.1 has an appeal in that we can visually and intuitively examine the similarities and dissimilarities between the nose pattern of each dog. However, if one wants to compare all possible pairs in Figure A1, the number of comparisons has to be 16,110, which is practically an impossible task. Moreover, visual examination, however intuitively appealing, is a qualitative comparison from which a sure-fire conclusion cannot be easily drawn. So, in order to come to a more scientifically sound conclusion, one has to rely on a more quantitative method of biometric technology.

We applied the template generation and matching methods described in Section 2.2 to calculate the matching distances of all 16,110 pairs of nose images, of which there are 180 genuine comparisons and 15,930 impostor comparisons. The result is summarized in Table 2 and Table 3.

Note that in Table 3, the maximum of the genuine matching distances is 0.3124, while the minimum of the impostor matching distances is 0.4175. This means that the matching distance between two nose images of the same dog is always smaller than that of any matching distance between two nose images of different dogs.

For convenience, let us take a number, say 0.4, and call it a threshold value, or in short, a threshold. To paraphrase what was said above, any genuine matching distance is less than this threshold, and any impostor matching distance is greater than this threshold.

This fact has a very important implication. Suppose one is presented with two nose images without knowing whether these images come from the same dog or from different dogs. Then, create the templates and do the matching as outlined in Section 2.2. If this matching distance is less than the threshold, i.e., 0.4, one declares that these two images come from the same dog; if, on the other hand, the matching distance is greater than the threshold, i.e., 0.4, one then declares that these two images come from different dogs.

This *decision process* produces no error, because the matching distance of any genuine pair has to be less than the threshold and the matching distance of any impostor pair has to be greater than the threshold. In other words, we have verified algorithmically the objectives spelled out at the end of Section 1, and hence that the nose pattern is an accurate and reliable biometric marker.

**Figure 5:**
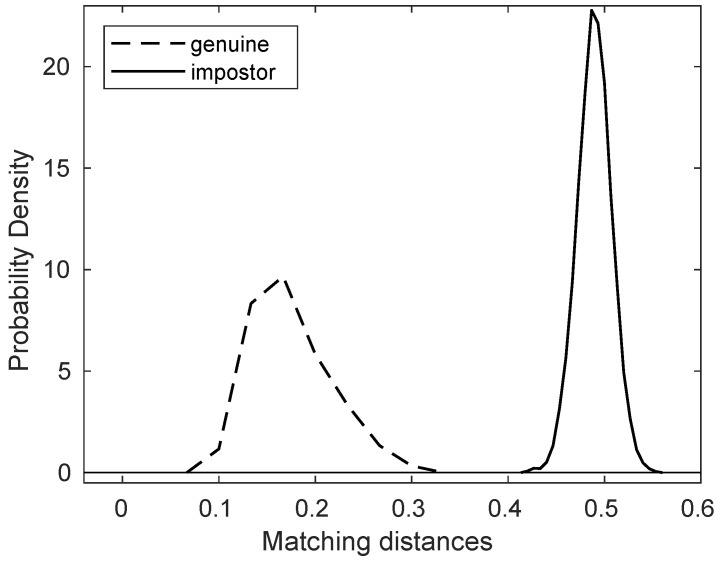
The probability distributions of genuine/impostor matching distances for the dataset in Figure A1.

Figure 5 also shows the graphs of the normalized histograms (probability distributions) of genuine and impostor matching distances. Note that the threshold value 0.4 separates these two graphs of probability distributions.

### 3.3. Algorithmic Verification II

We then enlarged the dataset by combining the image set of the 180 images in Figure A1 and 98 images collected for the work in the paper by Choi et al. [1]. We call this enlarged dataset the *combined dataset*. Note that this combined dataset contains 278 images of 70 dogs. The number of breeds involved in the combined dataset is 19, which is 18 breeds listed in Table 1 plus the beagle breed from the dataset used in [1].

Using this combined dataset, we again applied the template generation and matching methods described in Section 2.2 to calculate the matching distances of all 38,503 pairs of nose images, of which there are 612 genuine comparisons and 37,891 impostor comparisons. The result is summarized in Table 4 and Table 5.

The result of algorithmic verification for this combined dataset also leads us to the same conclusion as was drawn in Section 3.2.

Note again that in Table 5, the maximum of the genuine matching distances is 0.3577, while the minimum of the impostor matching distances is still 0.4175. As was observed in Section 3.2, we can see that the matching distance between two nose images of the same dog is always smaller than that of any matching distance between two nose images of different dogs. As was done in Section 3.2, let us take 0.4 as a threshold. Then with this threshold, we can also say, as was done in Section 3.2, that any genuine matching distance is less than this threshold and any impostor matching distance is greater than this threshold.

Using this threshold, we can apply the same *decision process* as described in Section 3.2. Namely, given two nose images without knowing their identities, one may compute the matching distance, and make the decision as follows: if the matching distance is less than the threshold, the two dogs have the same identity; if, on the other hand, it is greater than the threshold, the two dogs have different identities.

We have shown that this decision process produces no error; in other words, we have verified that our algorithm is capable of identifying the identity of dogs accurately without any error, which leads us to the conclusion that the canine nose pattern is unique to each individual dog and hence can be used as an accurate and reliable biometric marker.

**Figure 6:**
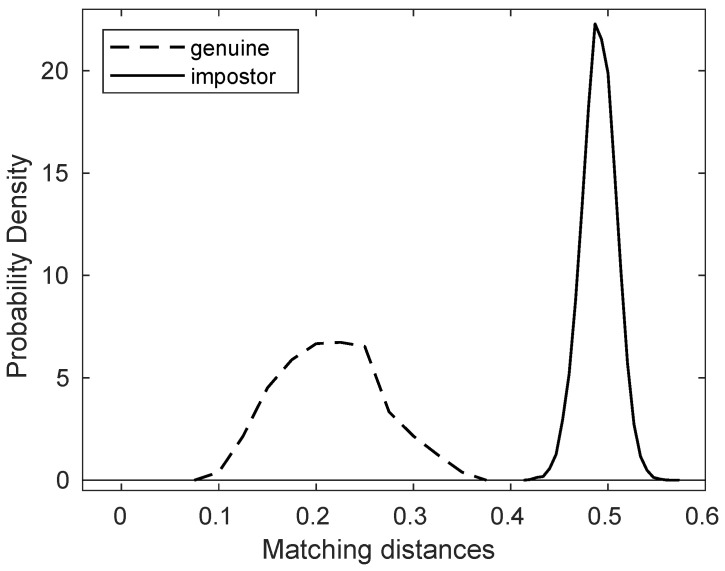
The probability distributions of genuine/impostor matching distances for the combined dataset.

Figure 6 also shows the graphs of the normalized histograms (probability distributions) of genuine and impostor matching distances. Note that the threshold value 0.4 separates these two graphs of probability distributions.

### 3.4. Discussion

This paper addresses the following fundamental question: “Is the canine nose pattern a unique biometric marker?” The earliest academic work in this regard was done by Horning et al. [2]. This work, though original and important, is mostly a statistical study. In a similar vein, Petersen [7] did a statistical study on the uniqueness of the bovine nose pattern. In comparison, our work delves deeper into the details of the canine nose pattern of each individual dog—first visually and then algorithmically to show that the canine nose pattern is indeed a unique biometric marker. Budras et al. [4] did a microscopic study to assert that “The surface pattern is individually specific and for this reason serves to identify the individual animal”, which also corroborates the claim of this paper on the uniqueness of the canine nose pattern.

Many more studies were done on the *algorithmic* aspects of biometric identification of cattle and pets. However, instead of focusing on the intrinsic nature of the canine (and bovine) nose pattern as we do in this paper, they are more interested in developing *workable* algorithms to be used in practice. As a result, all these results entail some error. However, it is not clear whether the error is due to algorithm or due to the intrinsic similarity of nose patterns of two different animals. It is, therefore, rather hard to draw a firm conclusion from these studies on the uniqueness of the nose pattern. In contrast, our work is specifically designed to answer the question of the uniqueness of canine nose pattern.

The work of Choi et al. [1] also addresses the same question of the uniqueness of canine nose pattern. However, the main focus of their work is to study the formation of the canine nose pattern. Therefore, as far as the uniqueness of canine nose patterns is concerned, their work has limited scope in that the data is only for one breed—the beagle dog, and the number of animals from which the data was collected is also small—ten. In comparison, this paper deals with a variety of breeds, age, gender, etc. The conclusion of the broader longitudinal cohort study of this paper provides a stronger and hence more convincing evidence that the canine nose pattern is indeed unique to each individual dog.

## 4. Conclusions

This paper shows that the canine nose pattern, which is an interlocking pattern of beads and grooves on a dog’s nose, is unique to each individual dog.

Our finding confirms and enhances the claims of earlier works by others that the canine nose pattern is unique to each animal and serves as a unique biometric marker.

This paper should be considered as a work complementing that of Choi et al. [1]. We believe both of them taken together provide even more convincing evidence that the canine nose pattern is indeed unique to each individual dog.

It should be emphasized that the primary concern of this paper is to study the intrinsic nature of the canine nose pattern; in particular, its uniqueness to each individual dog. Toward this goal, we have carefully collected *good* nose images to eliminate possible errors coming from bad or low quality images. This way, the test for the intrinsic nature of the uniqueness can be done more reliably. However, when it comes to practical matters like how to overcome possible errors and difficulties coming from low quality nose images, images taken at oblique angles, blurriness caused by a sudden head motion of a dog or even by shaking human hands, light reflected from the wet surface of a nose, etc., such are separate technical problems that are not the concern of this paper. Nonetheless, they are very important problems in practice that should be dealt with using good technology. We hope to be able to come back to these matters in the future.

## Figures and Tables

**Table 1:** The dates of image capture with details of each dog.

ID	Gender	Age in Years	Breed	Date (1st Batch)	Date (2nd Batch)	Date (3rd Batch)
01	F	5	Golden Retriever	22 September 2020	14 December 2020	5 April 2021
02	M	3	Golden Retriever	22 September 2020	14 December 2020	5 April 2021
03	F	4	Golden Retriever	22 September 2020	20 December 2020	10 April 2021
04	F	3	Bichon Frise	22 September 2020	19 December 2020	6 April 2021
05	F	2	Toy Poodle	22 September 2020	14 December 2020	7 April 2021
06	F	5	Toy Poodle	22 September 2020	17 December 2020	5 April 2021
07	M	2	Toy Poodle	22 September 2020	14 December 2020	7 April 2021
08	M	2	Bichon Frise	29 September 2020	23 December 2020	7 April 2021
09	F	7	Toy Poodle	22 September 2020	19 December 2020	6 April 2021
10	M	3	Pomeranian	22 September 2020	20 December 2020	5 April 2021
11	M	3	Maltese	22 September 2020	15 December 2020	9 April 2021
12	F	10	Maltese	24 September 2020	16 December 2020	8 April 2021
13	M	10	Shih Tzu	24 September 2020	19 December 2020	11 April 2021
14	M	3	Toy Poodle	24 September 2020	14 December 2020	6 April 2021
15	M	5	Toy Poodle	24 September 2020	17 December 2020	8 April 2021
16	M	5	Toy Poodle	24 September 2020	17 December 2020	8 April 2021
17	F	3	Golden Retriever	24 September 2020	17 December 2020	15 April 2021
18	M	6	Bichon Frise	24 September 2020	14 December 2020	5 April 2021
19	M	6	Toy Poodle	24 September 2020	16 December 2020	9 April 2021
20	M	6	Maltese	24 September 2020	16 December 2020	8 April 2021
21	M	5	Bichon Frise	26 September 2020	14 December 2020	5 April 2021
22	M	6	Shih Tzu	26 September 2020	19 December 2020	9 April 2021
23	M	4	Maltese	26 September 2020	19 December 2020	7 April 2021
24	M	2	Maltese	26 September 2020	14 December 2020	10 April 2021
25	M	4	Toy Poodle	26 September 2020	20 December 2020	11 April 2021
26	M	1	Pomeranian	26 September 2020	16 December 2020	6 April 2021
27	M	9	Maltese	26 September 2020	16 December 2020	10 April 2021
28	M	5	Toy Poodle	26 September 2020	20 December 2020	9 April 2021
29	M	4	Toy Poodle	26 September 2020	15 December 2020	10 April 2021
30	F	1	Pomeranian	26 September 2020	16 December 2020	9 April 2021
31	F	4	Toy Poodle	26 September 2020	14 December 2020	15 April 2021
32	M	3	Bichon Frise	26 September 2020	18 December 2020	10 April 2021
33	M	3	Toy Poodle	26 September 2020	19 December 2020	10 April 2021
34	F	3	Toy Poodle	26 September 2020	19 December 2020	10 April 2021
35	M	6	Maltese	29 September 2020	23 December 2020	8 April 2021
36	M	8	Toy Poodle	29 September 2020	14 December 2020	11 April 2021
37	F	5	Maltese	29 September 2020	19 December 2020	9 April 2021
38	M	7	Maltese	22 September 2020	14 December 2020	5 April 2021
39	M	2	Maltese	29 September 2020	21 December 2020	5 April 2021
40	F	6	Coton de Tulear	28 September 2020	18 December 2020	5 April 2021
41	F	3	Mixed-Breed Dog	28 September 2020	18 December 2020	5 April 2021
42	M	3	Standard Poodle	28 September 2020	18 December 2020	5 April 2021
43	M	8	Golden Retriever	25 September 2020	29 December 2020	19 April 2021
44	M	7	Border Collie	25 September 2020	29 December 2020	19 April 2021
45	M	3	Samoyed	25 September 2020	29 December 2020	19 April 2021
46	F	3	Samoyed	25 September 2020	29 December 2020	19 April 2021
47	F	6	Spitz	25 September 2020	29 December 2020	19 April 2021
48	F	7	Spitz	25 September 2020	29 December 2020	19 April 2021
49	F	5	Shetland Sheepdog	25 September 2020	29 December 2020	19 April 2021
50	F	7	Shetland Sheepdog	25 September 2020	29 December 2020	19 April 2021
51	F	7	Golden Retriever	25 September 2020	29 December 2020	19 April 2021
52	M	7	Samoyed	25 September 2020	29 December 2020	19 April 2021
53	F	2	Cocker Spaniel	25 September 2020	29 December 2020	19 April 2021
54	F	7	Welsh Corgi	25 September 2020	29 December 2020	19 April 2021
55	F	5	Welsh Corgi	25 September 2020	29 December 2020	19 April 2021
56	F	4	Welsh Corgi	25 September 2020	29 December 2020	19 April 2021
57	M	8	Miniature Pinscher	25 September 2020	29 December 2020	19 April 2021
58	M	1	Weimaraner	25 September 2020	29 December 2020	19 April 2021
59	M	8	Old English Sheepdog	25 September 2020	29 December 2020	19 April 2021
60	M	4	Toy Poodle	29 September 2020	18 December 2020	6 April 2021

**Table 2:** Comparison (matching) summary for the dataset in Figure A1.

# of Subjects	60
# of Images per subject	3
Total # of Images	180
# of Genuine comparisons	180
# of Impostor comparisons	15,930

**Table 3:** Matching distance statistics for the dataset in Figure A1.

Comparison Type	Min	Max	Mean	Std
genuine	0.0907	0.3124	0.1734	0.0416
impostor	0.4175	0.5551	0.4895	0.0180

**Table 4:** Comparison (matching) summary for the combined dataset.

# of Subjects	70
# of Images per subject	3, 9, or 10
Total # of Images	278
# of Genuine comparisons	612
# of Impostor comparisons	37,891

**Table 5:** Matching distance statistics for the combined dataset.

Comparison Type	Min	Max	Mean	Std
genuine	0.0907	0.3577	0.2150	0.0519
impostor	0.4175	0.5650	0.4905	0.0180

## Data Availability

All data used in the paper are displayed as images in Figure A1 in Appendix A. Higher resolution images may be available for the purpose of academic studies. Contact the corresponding author, Song-Hwa Kwon, at skwon@catholic.ac.kr for more details.

## Appendix A. Data

All image data used in this study are displayed in the Appendix section. Figure A1 shows all nose images and Figure A2 shows the corresponding ROI images. These ROI images are the original ROI images rotated and rescaled to make visual comparisons more intuitive and transparent.
